# Low genetic heterogeneity of *Leishmania major* in different geographical regions of Iran

**DOI:** 10.1371/journal.pone.0285520

**Published:** 2023-05-08

**Authors:** Adel Spotin, Soheila Rouhani, Ali Haghighi, Parviz Parvizi

**Affiliations:** 1 Immunology Research Center, Tabriz University of Medical Sciences, Tabriz, Iran; 2 Department of Parasitology and Mycology, School of Medicine, Tabriz University of Medical Sciences, Tabriz, Iran; 3 Department of Medical Parasitology and Mycology, School of Medicine, Shahid Beheshti University of Medical Sciences, Tehran, Iran; 4 Molecular Systematics Laboratory, Parasitology Department, Pasteur Institute of Iran, Tehran, Iran; Iran University of Medical Sciences, ISLAMIC REPUBLIC OF IRAN

## Abstract

To examine the genetic diversity of *Leishmania major*, 100 Giemsa-stained positive slides were collected from endemic foci of Iran (Northeast, Central, and Southwest provinces) over two consecutive years during 2019–2021. The *Leishmania* ITS-rDNA gene was amplified and *Leishmania* sp. was recognized by PCR-RFLP and sequencing. In addition, 178 registered ITS-rDNA sequences from other geographical regions of Iran were retrieved from GenBank, including different host species (human, sandfly and rodent). A total of 40 new haplotypes were discovered using the ITS-rDNA sequence analysis. IR29 (20.6%) and IR34 (61%) were the two most common haplotypes, represented by a star-like feature in the overall population. Analysis of the molecular variance test revealed low genetic diversity of *L*. *major* in human cases (Haplotype diversity; 0.341), rodent (Hd; 0.387) and sandfly (Hd; 0.390) sequences. The lowest genetic diversity of *L*. *major* was observed in Southwest/Southeast Iran (Hd: 0.104–0.286). The statistically *F*st value indicated that *L*. *major* is not genetically differentiated between geographic regions of Iran, except for the Northeast-Southwest (*F*st: 0.29055) and Central-Southwest (*F*st: 0.30294) population pairs. The current study as the first investigation discloses new perspectives for further evaluation in the identification local transmission paradigms and initiating effective prevention strategies.

## 1. Introduction

Cutaneous leishmaniasis (CL) is one of the neglected tropical diseases that is mainly spread in Iran by the causative agents of *Leishmania major* and *Leishmania tropica* [1, 2]. CL is prevalent in more than half of the Iranian provinces with a prevalence of 1.8% to 37.9% and an annual incidence of 26,630 cases/year [3, 4].

The spectrum of genetic (haplotype) variability in *L*. *major* is presented as one of the most controversial issues [1, 2, 5, 6]. The genetic heterogeneity of *Leishmania* species may result in the emergence of new species/strains/haplotypes and ultimately the creation of drug-resistant mutants [5, 6]. With regard to treatment failure, the identification of heterogeneity traits of *Leishmania* spp. and the emergence of resistant drug mutations should be addressed by policymakers.

Several mitochondrial and nuclear DNA markers, including microsatellites, ribosomal internal transcribed spacer regions (ITS-rDNA), cytochrome c, 18S-rRNA, HSP-70, the gp63 gene locus, and minicircles of kinetoplast DNA were used to assess the genetic diversity of the *Leishmania* parasite [1, 7–12]. The ITS-rDNA is a useful and informative phylogenetic marker as it has sequences with different rates of evolutionary variability [5, 13]. A number of molecular assays have been employed to identify genetic polymorphism between inter-and intra-*Leishmania* species [14, 15]. These include multilocus microsatellite typing (MLMT), random amplification of polymorphic DNA (RAPD), restriction fragment length polymorphism (RFLP), multilocus enzyme electrophoresis (MLEE), multilocus sequence typing (MLST) and sequencing.

Several Iranian researchers have performed their molecular experiments on *L*. *major* genotyping in different host species (human, sandfly and rodent) and their results have shown that the new haplotypes circulate sympatrically in endemic regions of the country [16–19]. However, according to our literature review, there is no comparative study on the genetic diversity of *L*. *major* among different endemic foci of Iran.

Knowledge of the genetic characteristics of *L*. *major* in adjacent regions may provide the basis for conducting future epidemiological studies to implement control strategies. The aim of this study was to assess the genetic diversity and gene migration of *L*. *major* using ITS-rDNA nucleotide sequences derived from human, sandfly and rodent in endemic regions of Iran.

## 2. Materials and methods

### 2.1. Ethics statement and sample collection

All research involving human participants have been obtained in conformity with informed consent, privacy and confidentiality of patients who were sampled and analyzed anonymously during study. The majorities of the people were in the edge of Iran border and therefore could not able to get written consent one by one. Instead, the oral consent was obtained with the help of central public health authority as both mutual acquaintance and interpreter of the indigenous peoples. All experiments on the humans were performed according to the guidelines of the Ethical Board of Pasteur Institute of Iran.

One hundred positive microscopic slides from wet/dry lesions of confirmed CL patients were collected from endemic geographical regions of Iran (Southwest, Khuzestan Province; n: 40, Northeast, Golestan Province; n: 30 and Central; Isfahan Province; n: 30) from August 2019 to February 2021. In this study, the collected samples from indigenous cases were included and the imported cases from neighboring countries were excluded.

The positive slides were graded to rank leishman bodies’ density from +1 to +6. In addition, in order to compare the analyzed sequences of the present study with five regions of Iran, 178 sequences of ITS-rDNA *L*. *major* including Southwest (Khuzestan, Ilam and Fars provinces), Central (Isfahan and Kashan provinces), North (Tehran Province), Southeast (Kerman), North-Central (Semnan), West (Lorestan and Kermanshah provinces) and Northeast (Golestan, Turkmen Sahra), Khorasan (Birjand, Esfarayen, Mashhad, Jajarm and Garmeh) populations were retrieved from the GenBank database for FASTA during 2008–2020.

### 2.2. DNA extraction, PCR amplification and PCR-RFLP for ITS-rDNA

DNA extraction of parasites was performed from Giemsa-stained positive slides (DNG Plus Kit, Iran) [20]. Single-round PCR was used to detect *Leishmania* sp. by amplifying ITS-rDNA about 480 bp. The details of PCR protocol and employed primers (ITS1F and ITS2R4) were the same as previously reported [1, 9, 17]. To digest the PCR amplicons in RFLP, the endonuclease reaction of ITS-rDNA was performed in a volume of 30μL containing, 2μL of 10x buffer, 2μL of *BsuRI* (*HaeIII)* (cut site GG↓CC), 10μL of amplicon and 16μL of distilled water for 4 h at 37^∘^C. The digested fragments were analyzed using electrophoresis on 1.5% agarose gel containing safe stain and ladder of 100 bp.

### 2.3. DNA sequencing, data extraction and bioinformatic analysis of ITS-rDNA

Forty PCR products were successfully sequenced using the ITS primers, ITS1F and ITS2R4 (Codon Company, Iran). Sequences were trimmed and edited in consensus positions compared to regional sequences using Sequencher v.5.4.6 software. Furthermore, 178 registered ITS-rDNA nucleotide sequences of *L*. *major* from other geographic populations of Iran including different host species (human, sandfly and rodent) were downloaded from GenBank. The analysis of molecular variance (AMOVA) was performed by DnaSP software to determine the genetic diversity indices (number of haplotypes (Hn); nucleotide diversity (Nd); haplotype diversity (Hd), neutrality indices (Tajima’s *D* and Fu’s Fs tests) [21]. The pairwise fixation index (*F*st: F-statistics) and number of migration (Nm) were obtained to assess the gene migration of *L*. *major* between different geographical regions of Iran. To ascertain the genealogical relationships of intra-species diversity of *L*. *major*, a haplotype network was drawn by PopART software determined by the Median-Joining model [22]. To confirm taxonomic status of the *L*. *major*, a phylogenetic tree was constructed using the program Splits Tree v.4.0 based on the Neighbor Net method and Median-Joining analysis. Distance scale was 0.01 indicating the number of base substitutions per site. Also *Trypanosoma brucei* (Accession number; **JN673390**) was considered as an outgroup branch.

## 3. Results

### 3.1. Identification of Leishmania sp. using PCR-RFLP

The clinical presentations of wet/dry lesions were classified into classic (volcanic form) and non-classic (psoriasiform, herpetiform, hyperkeratotic, eczematoid, zosteriform and erythematous papulonodules signs). The ITS-rDNA gene (480 bp) was amplified from all 100-CL stained slides. Based on the PCR-RFLP assay, all *Leishmania* PCR products were digested by *BsuRI (HaeIII)* and then two fragments of 140 bp and 340 bp were definitely assigned to *L*. *major*. To confirm the PCR-RFLP findings, 40 *L*. *major* amplicons were successfully sequenced, trimmed and edited to construct the phylogenetic tree.

### 3.2. Determination of genetic diversity and genetic differentiation between L. major

The edited sequences (n: 40) were compared with sequences obtained (n: 178) from the GenBank database. Ten sequences (including new haplotypes) were submitted to the GenBank database, and accession numbers were presented in S1 Table. A total of 40 new haplotypes were identified based on sequence analysis of the ITS-rDNA gene (S1 Table). According to AMOVA test, low genetic heterogeneity of *L*. *major* was found in Southwest (Hd: 0.104, Hn: 5), Southeast (Hd: 0.286, Hn: 2), Central (Hd: 0.419, Hn: 10), in the west (Hd: 0.334, Hn: 4), Northeast (Hd: 0.429, Hn: 17) and North-Central Iran (Hd: 0.400, Hn: 2) (Table 1).

**Table 1:** Diver sity and neutrality indices of *L*. *major* using ITS-rDNA sequences in various geographical regions of Iran.

Region Province/cities (n)		Diversity indices						Neutrality indices	
		N Hn Hd± SD Polymorphic sites Nd (π) K						Tajima’s D* Fu’s Fs statistic**	
**Central Iran**	Isfahan (16), Kashan (14) (Aran Bidgol)	30	10	0.419±0.080	17	0.01005	2.724	-1.49695	-9.012
**Southwest Iran**	Fars (1), Ilam (37), Khuzestan(10)	48	5	0.104±0.067	31	0.00894	2.083	-2.56931	4.546
**Northeast Iran**	Khorasan (Birjand; 25, Esfarayen; 31, Mashhad; 13, Jajarm; 5, Garmeh; 3), Golestan (37; Turkmen sahra)	114	17	0.429± 0.059	41	0.00901	2.459	-2.27541	-9.662
**Southeast Iran**	Kerman (n; 7, Bam, Jiroft and Shahdad)	7	2	0.286± 0.196	4	0.00351	1.143	-1.43414	2.047
**West Iran**	Lorestan (8), Kermanshah (6; Ghasre shirin)	14	4	0.334±0.045	29	0.02279	6.154	-1.75475	-0.796
**North central Iran**	Semnan (Damghan; 5)	5	2	0.400± 0.175	1	0.00202	0.600	1.22474	0.626
**Total**		218	40						

N: number of isolates; Hn: number of haplotypes; Hd: haplotype diversity; Nd: nucleotide diversity; K: Average number of nucleotide differences.*Significant, *P* < 0.01**Significant, *P* < 0.02

Based on host species, low genetic (haplotype) diversity of *L*. *major* was observed in reservoir rodents (*Rhombomys opimus* and *Meriones libycus*, Hd; 0.387, Hn: 10) and sandflies (*Phlebotomus papatasi/Phlebotomus caucasicus*, Hd; 0.390, Hn: 7) compared to human CL cases (Hd; 0.441, Hn: 23) (Table 2). The highest nucleotide diversity was found in Central Iran (Nd: 0.01005) particularly in reservoir rodents (Nd: 0.01164) (Tables 1 and 2). Neutrality indices of ITS-rDNA showed negative values (-2.27541 for Tajima’s D to -9.662 for Fu’s Fs statistic) in Northeast, West and Central populations which indicating a deviation from neutrality (Table 1). Within 480 bp consensus position of ITS-rDNA, 48 point mutations were identified. 24 of these were parsimony-informative sites and 24 of these were singleton variable sites. The statistically *F*st value showed that *L*. *major* is not genetically differentiated between geographic regions of Iran (*F*st: 0.01649 to 0.07820) except Northeast-Southwest (*F*st: 0.29055, Nm: 0.61) and Central-Southwest (*F*st: 0.30294, Nm: 0.58) population pairs (Table 3).

**Table 2:** Diversity and neutrality indices of *L*. *major* in different host species (human, sandfly and rodent) using ITS-rDNA sequences in various geographical regions of Iran.

Parasite Host-vector			Diversity indices						Neutrality indices	
			N Hn Hd± SD Nd (π) Polymorphic site K						Tajima’s D* Fu’s Fs statistic**	
***Leishmania major***	**Vector (Sandfly)**	***Phlebotomus papatasi/ Phlebotomus caucasicus***	25	7	0.390±0.079	0.00565	8	1.600	-0.77730	-1.466
	**Reservoir (Vertebrate hosts)**	**Human**	168	23	0.441 ± 0.030	0.00426	23	1.205	-2.03615	-29.979
		**Rodents: *Rhombomys opimus* and *Meriones libycus***	25	10	0.387± 0.105	0.01164	29	3.923	-1.95708	-2.662
**Total**			218	40						

* Significant, *P*<0.01

**Significant, *P* <0.02.

N: number of isolates; Hn: number of haplotypes; Hd: haplotype diversity; Nd: nucleotide diversity; K: Average number of nucleotide differences.

**Table 3:** Pairwise fixation index (*Fs*t) for *L*. *major* isolates originating from five regions of Iran using nucleotide sequence data of ITS-rDNA.

Region Province (cities)		Populations			
		Central Iran	Southwest Iran	West Iran	Northeast Iran
**Central Iran**	Isfahan and Kashan (Aran Bidgol)		Nm: 0.58	Nm: 6.52	Nm: 14.91
**Southwest Iran**	Fars, Ilam and Khuzestan	0.30294	-	Nm: 2.95	Nm: 0.61
**West Iran**	Lorestan, Kermanshah (Ghasre shirin)	0.03692	0.07820	-	Nm: 2.50
**Northeast Iran**	Khorasan (Birjand, Esfarayen, Mashhad, Jajarm and Garmeh), Golestan (Turkmen sahra)	0.01649	0.29055	0.09078	-

### 3.3. Phylogenetic tree and haplotype network of ITS-rDNA

**Fig 1:**
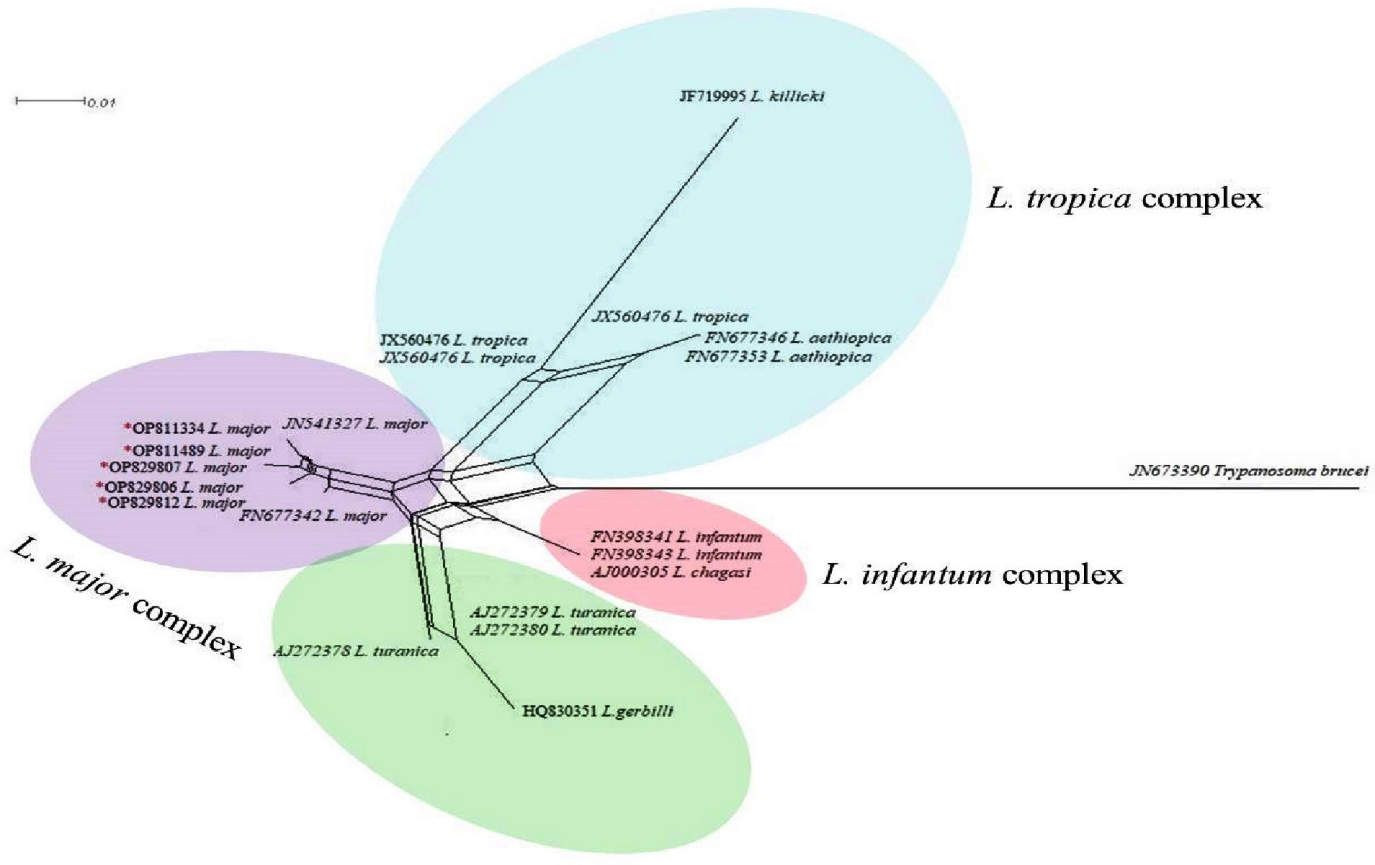
Neighbor-Net graph drawn by *Leishmania* spp. by using the Splits Tree 4.0 program. The identified isolates of *L*. *major* in this study marked by an asterisk (*). *Trypanosoma brucei* (Accession no: **JN673390**) was considered as out-group branch. The distance scale was estimated 0.01.

**Fig 2:**
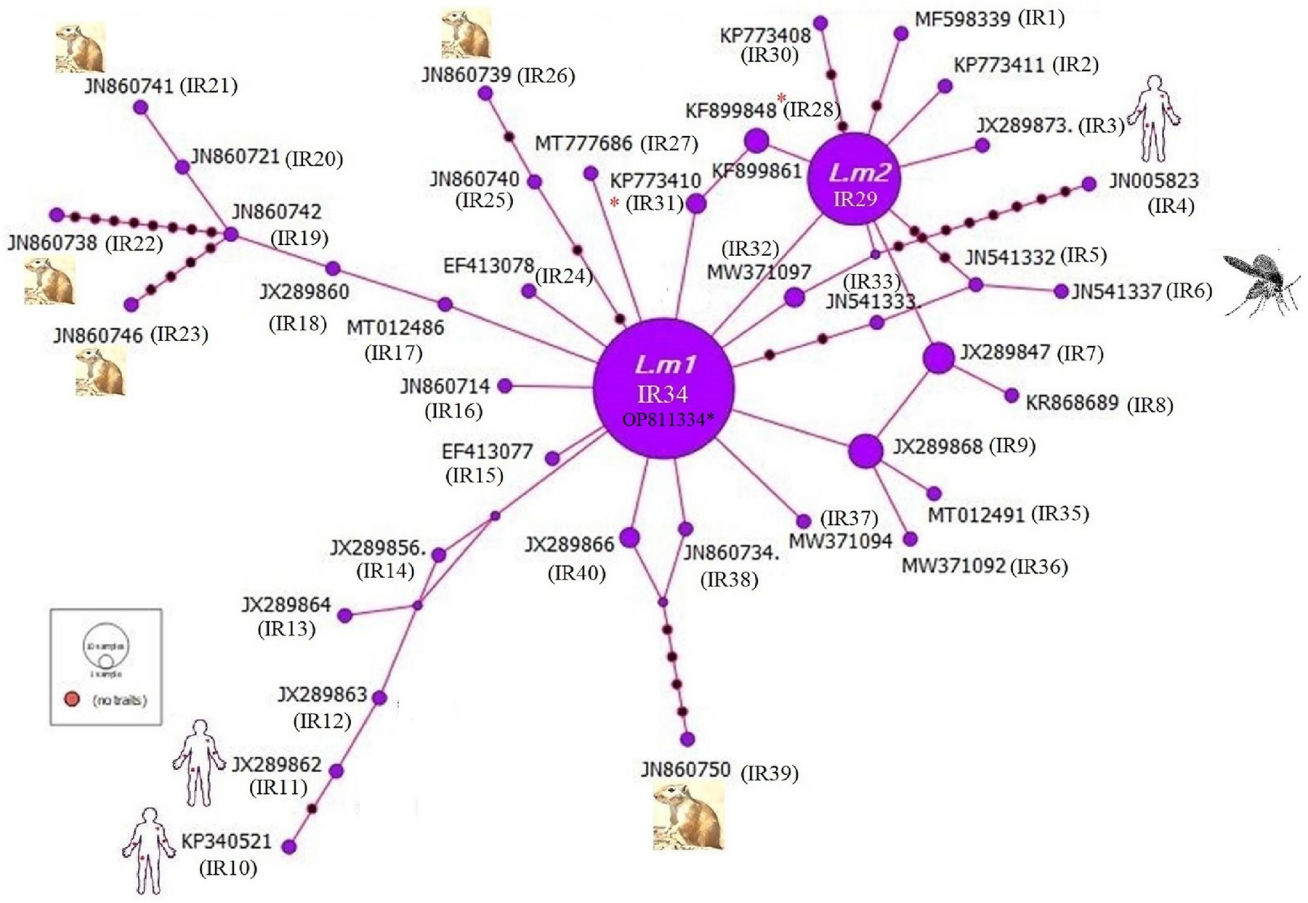
ITS-rDNA haplotype network in *L*. *major* from different host species (human, sandfly and rodent) in various geographical foci of Iran. Submitted sequences (n: 10) in this study were considered as common haplotype L. m1 (OP811334*). The red circles are relative to the frequency of each haplotype. Each line between haplotypes indicate single mutational step.

Neighbor Net tree generated by ITS-rDNA sequences demonstrated that *L*. *major* identified

(Accession nos; **OP811334, OP811489, OP811525, OP829807-OP829812**) assigned into *L*. *major* complex (Fig 1). The identified haplotypes (IR1-1R40) based on their different host species are presented in Fig 2. A total of 7, 10 and 23 haplotypes of *L*. *major* were detected in the sandfly, rodent and human, respectively in the different CL foci of Iran (Fig 2 and Table 2). IR29 (20.6%) and IR34 (61%) were the two most common haplotypes in the whole population, represented by a star-like feature in the overall population.

Occurrence of haplotypes of IR28 (Accession no: **KF899848**) and IR31 (Accession no: **KP773410**) between common haplotypes of IR29 and IR34 suggests that human-derived *L*. *major* may circulate between geographic regions of Iran (Fig 2).

## 4. Discussion

Knowledge of *L*. *major* genetic diversity of is essential to design epidemiological studies to implement monitoring, surveillance and control programs [5, 6]. This study analyzed the nucleotide sequence data on the heterogeneous population structure of *L*. *major* originating from sandfly, rodent and human hosts in Iran where different ecological conditions occur in widely separated regions.

In this study, the ITS-rDNA marker was used to detect the evolutionary relationship of *L*. *major*, as this DNA barcode marker can depict a reliable picture of the various phylogenetic subdivisions within this genus [13].

Based on current findings, micro-heterogeneity of *L*. *major* observed in reservoir rodents (*R*. *opimus* and *M*. *libycus*), sandfly vectors (*Ph*. *papatasi/Ph*. *caucasicus*) and human CL cases. This outcome indicates that rodents, sandflies, and human hosts are unlikely to exert a selective pressure on *L*. *major* and emphasizes the geographic/ecologic dependence of the low genetic heterogeneity in Iran. On the other hand, low genetic diversity of *L*. *major* in human, rodent and sandfly sequences can be explained by the genomic features of ITS-rDNA. In fact, the GC content of *L*. *major* (59.7%) is generally higher than that of *L*. *tropica*, so the stability of the triple hydrogen bonds of the GC pair and the stacking interaction are subjected to slippage [23, 24].

In a related study, low genetic heterogeneity of *L*. *major* isolates collected from patients and rodents was identified by MLMT technique in emergent foci of zoonotic cutaneous leishmaniasis in Tunisia [25, 26].

However, Tashakori et al., (2011) have shown three distinct genetic clusters of *L*. *major* (n: 26 isolates) using MLMT in Iran and they stated that significant genetic diversity of *L*. *major* is related to the existence of different populations of *Ph*. *papatasi* and/or differences in the abundance of reservoir hosts in different regions of Iran [27].

Some evidence suggests that the level of genetic diversity in the *Leishmania* parasite depends on the effective population size and is higher in small populations [5]. Furthermore, environmental alterations and biotic interactions among five regions of Iran can exert strong selective pressures on different life-history aspects and hence influence their degree of genetic diversity in *Leishmania* parasite [5]. However, other authors have reported a decreasing degrees of genetic diversity among the following *Leishmania* spp.; *L*. *tropica*> *L*. *aethiopica*> ***L*. *major***> *L*. *donovani* [28].

In this study, a variety of clinical manifestations (classical and non-classical forms) of *L*. *major* were observed in human lesions, however no significant genetic diversity (Hd; 0.541) was found among ITS-rDNA sequences of *L*. *major* in Khuzestan, Southwest Iran. These findings attenuate relationship between phenotypic and genotypic traits of CL in *L*. *major* patients, possibly demonstrating that genetic heterogeneity may not definitely influence the formation of various clinical manifestations.

The negative neutrality indices for the *L*. *major* population indicate evidence of some likely mechanisms including the neutral mutation model, population size equilibrium and purifying selection [29].

The significant *F*st values (0.29055 to 0.30294) of ITS-rDNA sequences showed that *L*. *major* Northeast-Southwest and Central-Southwest populations were genetically differentiated. It could be postulated that no gene migration of *L*. *major* taken place between local isolates of the mentioned regions. This also indicates that the diversity of the *L*. *major* population was unlikely to be affected by the bottleneck effect.

Furthermore, the occurrence of distinct genetic structure of *L*. *major* between mentioned regions indicates that this protozoan may have been sustained by indigenous human, rodents and sandflies during the era. On the other hand, low values of *F*st (0.01649 to 0.07820) showed that *L*. *major* West-Central, Central-Northeast, West-Southwest populations were not genetically differentiated, indicating a gene migration of *L*. *major* has probably occurred between mentioned population pairs.

The emergence of haplotypes of IR28 (human isolate) and IR31 (human isolate) between common haplotypes of IR29 and IR34 shows that here is dawn of *L*. *major* migration as a result of transmission of haplotypes from one population to another population through ecological changes and/or host mobility.

Charyyeva et al., (2021) demonstrated that *L*. *tropica* isolated from Syrian and Turkish patients using ITS1 sequences exhibits a complex phylo-geographic pattern, with some haplotypes being widespread across the Turkey and Syria [30].

One of the limitations of the current study was that the *L*. *major* sequences obtained from sandflies and rodents were partially small, in order to infer the extensive genetic diversity on a large scale.

In conclusion, the present study as the first investigation strengthens our knowledge of local transmission paradigms and the genetic data of *L*. *major* in different geographical regions of Iran. Moreover low genetic heterogeneity of *L*. *major* in human, rodent and sandfly hosts should be highlighted as a treatment target for the emergence of probable drug-resistant mutants, particularly in clinical *Leishmania* strains. Further research is needed to develop next-generation sequencing of *Leishmania* sp. through the use of informative markers in larger areas of Iran and surrounding countries to assess the potential evolutionary scenario.

## Supporting information

S1 Table Identified haplotypes of *L*. *major* based on ITS-rDNA sequences in various geographical foci of Iran. (DOCX)

## References

[1] Spotin A, Rouhani S, Parvizi P (2014) The associations of *Leishmania major* and *Leishmania tropica* aspects by focusing their morphological and molecular features on clinical appearances in Khuzestan province, Iran. *BioMed Res Int* 2014. 10.1155/2014/913510 PMC4181938 25317423

[2] Mirzapour A, Spotin A, Behniafar H, Azizi H, Maleki B, et al. (2019) Intra-species diversity of *Leishmania major* and *L*. *tropica* from clinical isolates of cutaneous leishmaniasis in southwest iran inferred by ITS1-rDNA. *Iran J Public Health* 48: 893. 31523646 PMC6717422

[3] Alvar J, Vélez ID, Bern C, Herrero M, Desjeux P, et al. (2012) Leishmaniasis worldwide and global estimates of its incidence. *PLoS One* 7: e35671. doi: 10.1371/journal.pone.0035671 22693548 PMC3365071

[4] Badirzadeh A, Asadgol Z, Spotin A, Mokhayeri Y, Shirzadi M, et al. (2015) The burden of leishmaniasis in Iran: findings from the global burden of disease from 1990 to 2010; Asian Pac J Trop Dis 7(9):513–518.

[5] Banuls A-L, Hide M, Prugnolle F (2007) *Leishmania* and the leishmaniases: a parasite genetic update and advances in taxonomy, epidemiology and pathogenicity in humans. *Adv Parasitol* 64: 1–458. 17499100 10.1016/S0065-308X(06)64001-3

[6] Santi AMM, Murta SMF (2022) Impact of Genetic Diversity and Genome Plasticity of *Leishmania* spp. in Treatment and the Search for Novel Chemotherapeutic Targets. *Front Cell Infect Microbiol* 9. 10.3389/fcimb.2022.826287 PMC8819175 35141175

[7] Yatawara L, Le TH, Wickramasinghe S, Agatsuma T (2008) Maxicircle (mitochondrial) genome sequence (partial) of *Leishmania major*: gene content, arrangement and composition compared with *Leishmania tarentolae*. Gene 424: 80–86. 18718512 10.1016/j.gene.2008.07.026

[8] Yurchenko V, Kolesnikov AA, Lukes J (2000) Phylogenetic analysis of Trypanosomatina (Protozoa: Kinetoplastida) based on minicircle conserved regions. *Folia Parasitol* 47: 1–5. doi: 10.14411/fp.2000.001 10833008

[9] Parvizi P, Ready P (2008) Nested PCRs and sequencing of nuclear ITS‐rDNA fragments detect three *Leishmania* species of gerbils in sandflies from Iranian foci of zoonotic cutaneous leishmaniasis. Trop Med Int Health 13: 1159–1171. 18631311 10.1111/j.1365-3156.2008.02121.x

[10] Montalvo A, Fraga J, Monzote L, Montano I, De Doncker S, et al. (2010) Heat-shock protein 70 PCR-RFLP: a universal simple tool for *Leishmania* species discrimination in the New and Old World. Parasitology 137: 1159–1168. 20441679 10.1017/S0031182010000089

[11] Bulle B, Millon L, Bart J-M, Gállego M, Gambarelli F, et al. (2002) Practical approach for typing strains of *Leishmania infantum* by microsatellite analysis. *J Clin Microbiol* 40: 3391–3397. 12202583 10.1128/JCM.40.9.3391-3397.2002 PMC130734

[12] Victoir K, Banuls A, Arevalo J, Llanos-Cuentas A, Hamers R, et al. (1998) The gp63 gene locus, a target for genetic characterization of *Leishmania* belonging to subgenus Viannia. Parasitology 117: 1–13. 9695095

[13] Hillis DM, Moritz C, Porter CA, Baker RJ (1991) Evidence for biased gene conversion in concerted evolution of ribosomal DNA. Science 251: 308–310. doi: 10.1126/science.1987647 1987647

[14] Schönian G, Kuhls K, Mauricio I (2011) Molecular approaches for a better understanding of the epidemiology and population genetics of *Leishmania*. Parasitology 138: 405–425. 21078222 10.1017/S0031182010001538

[15] Spotin A, Rouhani S, Ghaemmaghami P, Haghighi A, Zolfaghari MR, et al. (2015) Different morphologies of *Leishmania major* amastigotes with no molecular diversity in a neglected endemic area of zoonotic cutaneous leishmaniasis in Iran. *Iran Biomed J* 19: 149. 10.7508/ibj.2015.03.004 PMC4571010 26081070

[16] Oryan A, Shirian S, Tabandeh M-R, Hatam G-R, Randau G, et al. (2013) Genetic diversity of *Leishmania major* strains isolated from different clinical forms of cutaneous leishmaniasis in southern Iran based on minicircle kDNA. *Infect Genet Evol* 19: 226–231. 23892374 10.1016/j.meegid.2013.07.021

[17] Sharbatkhori M, Spotin A, Taherkhani H, Roshanghalb M, Parvizi P (2014) Molecular variation in *Leishmania* parasites from sandflies species of a zoonotic cutaneous leishmaniasis in northeast of Iran. *J Vector Borne Dis* 51: 16–21. 24717197

[18] Mirzaei A, Rouhani S, Taherkhani H, Farahmand M, Kazemi B, et al. (2011) Isolation and detection of Leishmania species among naturally infected Rhombomis opimus, a reservoir host of zoonotic cutaneous leishmaniasis in Turkemen Sahara, North East of Iran. *Exp Parasitol* 129: 375–380. doi: 10.1016/j.exppara.2011.08.020 21945269

[19] Hajjaran H, Mohebali M, Mamishi S, Vasigheh F, Oshaghi MA, et al. (2013) Molecular identification and polymorphism determination of cutaneous and visceral leishmaniasis agents isolated from human and animal hosts in Iran. BioMed Res Int 2013. doi: 10.1155/2013/789326 24286085 PMC3826333

[20] Parsaei M, Raeisi A, Spotin A, Shahbazi A, Mahami-Oskouei M, et al. (2018) Molecular evaluation of pvdhfr and pvmdr-1 mutants in *Plasmodium vivax* isolates after treatment with sulfadoxine/pyrimethamine and chloroquine in Iran during 2001–2016. *Infect Genet Evol* 64: 70–75. 29929007 10.1016/j.meegid.2018.06.021

[21] Rozas J, Sánchez-DelBarrio JC, Messeguer X, Rozas R (2003) DnaSP, DNA polymorphism analyses by the coalescent and other methods. Bioinformatics 19: 2496–2497. doi: 10.1093/bioinformatics/btg359 14668244

[22] Bandelt H-J, Forster P, Röhl A (1999) Median-joining networks for inferring intraspecific phylogenies. Mol Biol Evol 16: 37–48. doi: 10.1093/oxfordjournals.molbev.a026036 10331250

[23] Ivens AC, Peacock CS, Worthey EA, Murphy L, Aggarwal G, et al. (2005) The genome of the kinetoplastid parasite, *Leishmania major*. Science 309: 436–442. 16020728 10.1126/science.1112680 PMC1470643

[24] Schönian G, Schnur L, El Fari M, Oskam L, Kolesnikov AA, et al. (2001) Genetic heterogeneity in the species *Leishmania tropica* revealed by different PCR-based methods. *Trans R Soc Trop Med Hyg* 95: 217–224. 11355565 10.1016/s0035-9203(01)90173-7

[25] Attia H, Sghaier RM, Gelanew T, Bali A, Schweynoch C, et al. (2016) Genetic micro-heterogeneity of *Leishmania major* in emerging foci of zoonotic cutaneous leishmaniasis in Tunisia. *Infect Genet Evol* 43: 179–185. 27137082 10.1016/j.meegid.2016.04.032

[26] Ghawar W, Attia H, Bettaieb J, Yazidi R, Laouini D, et al. (2014) Genotype profile of *Leishmania major* strains isolated from tunisian rodent reservoir hosts revealed by multilocus microsatellite typing. *PLoS One* 9: e107043. 25203305 10.1371/journal.pone.0107043 PMC4159323

[27] Mahnaz T, Al-Jawabreh A, Kuhls K, Schönian G (2011) Multilocus microsatellite typing shows three different genetic clusters of *Leishmania major* in Iran. Microbes Infect 13: 937–942. 21664984 10.1016/j.micinf.2011.05.005

[28] Schönian G, El Fari M, Lewin S, Schweynoch C, Presber W (2001) Molecular epidemiology and population genetics in *Leishmania*. Med Microbiol Immunol 190: 61–63. 11770112 10.1007/s004300100081

[29] Huyse T, Poulin R, Theron A (2005) Speciation in parasites: a population genetics approach. Trends Parasitol 21: 469–475. doi: 10.1016/j.pt.2005.08.009 16112615

[30] Charyyeva A, Çetinkaya Ü, Özkan B, Şahin S, Yaprak N, et al. (2021) Genetic diversity of *Leishmania tropica*: Unexpectedly complex distribution pattern. Acta Trop 218: 105888. 33713626 10.1016/j.actatropica.2021.105888

