# Supplementary material for: Low genetic heterogeneity of Leishmania major in different geographical regions of Iran
Source: PLoS One. 2023 May 8;18(5):e0285520. doi: 10.1371/journal.pone.0285520 (PMC10166485; doi:10.1371/journal.pone.0285520)
Supplement: S1 Table — (DOCX) [file pone.0285520.s001.docx]

| **Haplotype ID.**  Supplementary 1 Table: Identified haplotypes of *L. major* based on ITS-rDNA sequences in various geographical foci of Iran. | **Country/Province/City** | | **Host**  **(** **Reservoir/Vector)** | **Accession numbers** |
| --- | --- | --- | --- | --- |
| IR1 | Iran/Kerman/Sirjan | Human | | MF598339 |
| IR2 | Iran/Ilam/Dehloran | Human | | KP773411 |
| IR3 | Iran/Ilam/Dehloran | Human | | JX289873 |
| IR4 | Iran/Tehran | Human | | JN005823 |
| IR5 | Iran/South  khorasan/Birjand | Sandfly  *(Phlebotomus caucasicus)* | | JN541332 |
| IR6 | Iran/Birjand | Sandfly  *(Phlebotomus caucasicus)* | | JN541337 |
| IR7 | Iran/Bam | Human | | JX289847 |
| IR8 | Iran/Yazd | Human | | KR868689 |
| IR9 | Iran/Kermanshah | Human | | JX289868 |
| IR10 | Iran/Ilam/Dehloran | Human | | KP340521 |
| IR11 | Iran/Isfahan | Human | | JX289862 |
| IR12 | Iran/Isfahan | Human | | JX289863 |
| IR13 | Iran/Isfahan | Human | | JX289864 |
| IR14 | Iran/Isfahan | Human | | JX289856 |
| IR15 | Iran/Turkmen Sahra | Sandfly  *(Phlebotomus papatasi)* | | EF413077 |
| IR16 | Iran/Ilam/Dehloran | Human | | JN860714 |
| IR17 | Iran/North khorasan | Human | | MT012486 |
| IR18 | Iran/Isfahan | Human | | JX289860 |
| IR19 | Iran/Golestan | Rodent  *(Rhombomys opimus)* | | JN860742 |
| IR20 | Iran/Kermanshah | Human | | JN860721 |
| IR21 | Iran/Golestan | Rodent  *(Rhombomys opimus)* | | JN860741 |
| IR22 | Iran/Golestan | Rodent  *(Rhombomys opimus)* | | JN860738 |
| IR23 | Iran/Golestan | Rodent  *(Rhombomys opimus)* | | JN860746 |
| IR24 | Iran/Turkmen Sahra | Sandfly  *(Phlebotomus papatasi)* | | EF413078 |
| IR25 | Iran/Golestan | Rodent  *(Rhombomys opimus)* | | JN860740 |
| IR26 | Iran/Golestan | Rodent  *(Rhombomys opimus)* | | JN860739 |
| IR27 | Iran/North  khorasan/Jajarm | Human | | MT777686 |
| IR28 | Iran/Ilam/Dehloran | Human | | KF899848 |
| IR29 (20.6%)  (***L. m2***)  Common haplotype | Iran/Ilam/Dehloran | Human | | KF899861 |
| IR30 | Iran/Ilam/Mehran | Human | | KP773408 |
| IR31 | Iran/Ilam/Mehran | Human | | KP773410 |
| IR32 | Iran/North  khorasan/Jajarm | Human | | MW371097 |
| IR33 | Iran/Sorth  khorasan/JBirjand | Sandfly  *(Phlebotomus papatasi)* | | JN541333 |
| IR34 (61%)  (***L. m1***)  Common haplotype | **Current Study**; Iran; Isfahan, Golestan and Khuzestan provinces | Human | | **OP811334***  **OP811489***  **OP811525***  **OP829807***  **OP829809***  **OP829806***  **OP829808***  **OP829812***  **OP829811***  **OP829810*** |
| IR35 | Iran/North  khorasan/Jajarm | Human | | MT012491 |
| IR36 | Iran/North khorasan/  Esfarayen | Human | | MW371092 |
| IR37 | Iran/North khorasan/  Garmeh | Human | | MW371094 |
| IR38 | Iran/Khorasan | Rodent  *(Rhombomys opimus)* | | JN860734 |
| IR39 | Iran/Golestan | Rodent  *(Rhombomys opimus)* | | JN860750 |
| IR40 | Iran/Isfahan | Human | | JX289866 |
