# Supplementary material for: Low genetic heterogeneity of Leishmania major in different geographical regions of Iran
Source: PLoS One. 2023 May 8;18(5):e0285520. doi: 10.1371/journal.pone.0285520 (PMC10166485; doi:10.1371/journal.pone.0285520)
Supplement: Attachment — Submitted filename: R1 point-by-point response to reviewers 11 March 2023.docx [file pone.0285520.s002.docx]

**Dear Dr. Alireza Badirzadeh**

**This is my FIRST revision of manuscript**

**Ms. Ref. No.: PONE-D-23-00609**

**Title: “Low genetic heterogeneity of *Leishmania major* in various geographical regions of Iran”**

**Reviewers' comments:

Reviewer's Responses to Questions**

**Comments to the Author**

Reviewer #1: Authors investigated the genetic diversity of Leishmania major, 100 positive stained slides were collected from endemic foci of Iran (Northeastern, Central, and Southwestern provinces) over two consecutive years during 2019-2021. In general this is well-written manuscript, important for the field and for the region. I suggest to cut Introduction and to focus only on the topic, also please be focused in Discussion. Methodology is well written. English should be revised by English speaker.

Response: Thank you. Discussion was revised and English was polished entire MS.

Reviewer #2: The manuscript presents a good concept; however, some points are not clear to the reviewer which are summarized below:
In Abstract
-In line 15: “100 positive stained slides” please determine, Giemsa or Wright stained slides?

Response: The “100 Giemsa stained positive” was highlighted in line15.
-In line 24, please correct the spelling of "Haplotype". And check the spelling of all of the text.

Response: It was done and highlighted in line 24.

-Please “PCR-RFLP” added to the Keywords. Response: It was done and highlighted
In introduction
-Considering the low genetic diversity of L. major was observed in other studies in Iran, please explain the novelty and necessity of conducting this study.

Response: In this study, for the first time, haplotype distribution and gene migration of *Leishmania major* were assessed among various sources of human, rodent and sandfly in different geographical regions of Iran which can depict a real picture of epidemiology of parasite.

-In lines 49-53: Despite the existence of a number of molecular assays for identifying genetic polymorphism, why did you choose the "RFLP" method among them? Response: Among the known molecular methods, the PCR-RFLP for ITS-rDNA using BsuRI (HaeIII) enzyme has widely used because of the ease in differentiating *Leishmania major, L. tropica and L. infantum*.

In Materials and methods
-According to the wideness of the selected geographical areas, the number of 100 slides is not suitable. Was there a reason for not providing more slides?

Response: In this study, we were able to collect 100 positive slides during 2019-2021. To solve this gap, the sequences recorded (GenBank) from Iran was used to compare genomic analyzes.

-What inclusion and exclusion criteria are considered in the selection of patients? For example, are immigrants or imported cases considered?

Response: In this study, the collected samples from indigenous cases were included and the imported cases from neighboring countries were excluded (Lines 67-68).

-In lines 65 and 86: Please write the number in the form of a letter at the beginning of the sentence, for example, "one hundred". Response: Thank you. It was done and highlighted.

In discussion
-In line 148: the full form of ITS-rDNA has already been mentioned, please just write the abbreviation form. Response: It was done and highlighted.

-Please mention the limitations of this research in the discussion section. Response: It was mentioned in line 193.

Reviewer #3: The manuscript entitled "Low genetic heterogeneity of Leishmania major in various geographical regions of Iran" describes the diversity of L. major isolates and its implication on the epidemiology of zoonotic cutaneous leishmaniasis in Iran. However, several points must be addressed. Thus, I recommend that this manuscript should be published only after considering these major following changes.

Specific Comments
- All the Tables can go to supplements. Response: Due to the fact that tables 2 to 4 are directly related to the explanations of the results section of the article, therefore table 1 was considered as supplementary file.
- Please explain why only 40 amplicons (out of 100) were sequenced? Response: In this study, only 40 samples were successfully sequenced due to limited financial resources, bad sequencing and permanent non-specific bands.
- Please put your research in the context of similar studies in other Leishmania spp. from the same region. Response: It was done in conclusion and highlighted.
- The authors should discuss further the results obtained by this method used in this study compared to the findings of other studies performed by using MLMT or NGS reporting contrasting results. Response: It was done (lines 161-163, 164-167).
- The authors referred to one study instead to reporting the results of most studies performed on L. major. Response: It was discussed and highlighted in discussion.

- The authors should discuss the findings of other studies performed in Turkey and Syria (entitled: Genetic diversity of Leishmania tropica: Unexpectedly complex distribution pattern; F.e. doi:10.1016/j.actatropica.2021.105888.) by comparison with the results of this study.

Response: It was discussed and highlighted in lines 194-196.

- - Resolution of figures must be improved. Response: The resolution of Figs was improved to 300 dpi.

- Line 68: The location of Fig 2 don’t support in the text. Response: Thank you. It was corrected.

**Kind Regards**

**Adel Spotin, Ph.D.**

***Corresponding author:** **Immunology Research Center, Tabriz University of Medical Sciences, Tabriz, Iran, Email:** [**Adelespotin@gmail.com**](mailto:Adelespotin@gmail.com)**,** [**Spotina@tbzmed.ac.ir**](mailto:Spotina@tbzmed.ac.ir)**.**
